# Source apportionment of groundwater pollutants in Apulian agricultural sites using multivariate statistical analyses: case study of Foggia province

**DOI:** 10.1186/1752-153X-6-S2-S5

**Published:** 2012-05-02

**Authors:** Pierina Ielpo, Daniela Cassano, Antonio Lopez, Giuseppe Pappagallo, Vito Felice Uricchio, Pasquale Abbruzzese De Napoli

**Affiliations:** 1 Water Research Institute – National Research Council, via F. De Blasio, 5 – 70132 Bari, Italy

## Abstract

**Background:**

Ground waters are an important resource of water supply for human health and activities. Groundwater uses and applications are often related to its composition, which is increasingly influenced by human activities.

In fact the water quality of groundwater is affected by many factors including precipitation, surface runoff, groundwater flow, and the characteristics of the catchment area. During the years 2004-2007 the Agricultural and Food Authority of Apulia Region has implemented the project “Expansion of regional agro-meteorological network” in order to assess, monitor and manage of regional groundwater quality. The total wells monitored during this activity amounted to 473, and the water samples analyzed were 1021. This resulted in a huge and complex data matrix comprised of a large number of physical-chemical parameters, which are often difficult to interpret and draw meaningful conclusions. The application of different multivariate statistical techniques such as Cluster Analysis (CA), Principal Component Analysis (PCA), Absolute Principal Component Scores (APCS) for interpretation of the complex databases offers a better understanding of water quality in the study region.

**Results:**

Form results obtained by Principal Component and Cluster Analysis applied to data set of Foggia province it’s evident that some sampling sites investigated show dissimilarities, mostly due to the location of the site, the land use and management techniques and groundwater overuse. By APCS method it’s been possible to identify three pollutant sources: Agricultural pollution 1 due to fertilizer applications, Agricultural pollution 2 due to microelements for agriculture and groundwater overuse and a third source that can be identified as soil run off and rock tracer mining.

**Conclusions:**

Multivariate statistical methods represent a valid tool to understand complex nature of groundwater quality issues, determine priorities in the use of ground waters as irrigation water and suggest interactions between land use and irrigation water quality.

## Background

Ground water serves a number of important functions for humanity and nature. These functions are often related to groundwater composition, which is increasingly influenced by human activities. To assess whether ground water will maintain its present function in future, it’s necessary to obtain insight into the factors determining groundwater composition.

In fact the groundwater quality is affected by many factors including precipitation, surface runoff, groundwater flow, and the characteristics of the catchment area. In particular groundwater composition is determined by initial water composition during infiltration, by groundwater flow patterns and by characteristics of the aquifer. The initial water composition is primarily related to the origin of the recharge water, e.g. precipitation or surface water. During infiltration, changes in water composition may occur through natural processes or through human activities dependent on soil conditions and land use (e.g. evapotranspiration and dissolution of fertilizers). Flow patterns determine the spatial displacement of ground water and dissolved solids through the subsurface. Groundwater flow depends on natural factors (e.g. elevation differences and lithology) and on human interventions (e.g. groundwater extraction and drainage).

The relative water levels between the groundwater and polluted surface waters determine the amount and nature of the deterioration in the groundwater quality [1,2].

During the years 2004-2007 the Agricultural and Food Authority of Apulia Region has implemented the project “Expansion of regional agro-meteorological network” in order to assess, monitor and manage of regional groundwater quality. The wells monitored during this activity amounted to 473, and the water samples analyzed were 1021.

This resulted in a huge and complex data matrix comprised of a large number of physical-chemical parameters, which are often difficult to interpret and draw meaningful conclusions. Further, for effective pollution control and water resource management, it is required to identify the pollution sources and their quantitative contributions [3,4]. Traditional approaches to assessing water quality are based on the comparison of experimentally determined parameter values with the existing guidelines but in many cases it does not readily give information on status of the source [5].

The application of different multivariate statistical techniques such as cluster analysis, principal component analysis, source apportionment by multiple linear regression on absolute principal component scores for interpretation of the complex databases offers a better understanding of water quality in the study region.

In fact advantages of multivariate statistical techniques for environmental data can be summarised as:

• reflect more accurately the multivariate nature of natural ecological system

• provide a way to handle large data sets with large numbers of variables by summarizing the redundancy

• provide a means of detecting and quantifying truly multivariate patterns that arise out of the correlation structure of the variable set [6].

These techniques also permit identification of the possible factors/sources that are responsible for the variations in water quality and influence the water system and in apportionment of the sources, which, thus offer valuable tool for developing appropriate strategies for effective management of the water resources [7-12].

In the present paper, the results obtained from monitoring activity performed in Foggia district (one of the Apulian provinces located in the North part of Apulia region) during the years 2004-2007 in the frame of the project “Expansion of the Regional Agro-meteorological Monitoring Network” are shown. In fact the Agriculture and Food Authority of Apulia Region, in partnership with the Regional Farmer Consortium (Asso.Co.Di. Puglia), CNR-IRSA and Bari University, in 2004 has launched a Water and Soil Monitoring Campaign for the purpose of checking the quality of soils and ground waters, used for irrigation, and then the quality level of the regional agricultural produces. This Project also was aimed to support the farmers to adopt the Best Management Practices (*BMPs*) and to reduce the water consume and the power and chemical (nutrients and pesticides) inputs in agriculture.

In table 1 and table 2 the main crops [13] with relative extension in Apulia region and Foggia province are respectively summarized.

**Table 1:** Main crops in Apulia region

CROP	Hectares (Ha)	% (regional area)
CEREALS	650,000	35.0
OLIVE GROVES	420,000	22.0
VINEYARDS / ORCHARDS	320,000	17.0
FORESTS	100,000	5.0
PASTURE	90,000	4.5

**Table 2:** Main crops in Foggia province

CROP	Hectares (Ha)	% (provincial area)
CEREALS cereals/tomatoes/vegetables (rotation)	350,000	50
FORESTS	80,000	11
OLIVE GROVES	45,000	6.5
VINEYARDS / ORCHARDS	45,000	6.5
PASTURE	35,000	5

The Project founded on a tight soil and water sampling collection, carried out all around the region, and on the determination of the main physical and chemical parameters of soils and waters.

The large data base was subjected to different multivariate statistical techniques with a view to extract information about the similarities or dissimilarities among the sampling sites, identification of water quality variables responsible for spatial and temporal variations, the influence of the possible sources (natural and anthropogenic) on the water quality parameters and the source apportioning for estimation of the contribution of possible sources on the concentration of determined water quality parameters of ground waters of Foggia province.

## Results and discussion

In table 3 descriptive statistics of groundwater variables collected in Foggia district during the years 2004-2007 are shown. As one can see some parameters show very high variance. This reflect the fact that environmental data are affected the wide variety of natural and anthropogenic influences.

**Table 3:** Descriptive statistics of Foggia province groundwater variables

Parameter	Average	Median	Mode	Standard deviation	Minimum	Maximum
pH	7,22	7,18	7,00	0,24	6,79	7,93
Electrical conductivity (mS/cm)	2,74	1,93	3,40	2,02	0,46	10,34
Total dissolved solids (TDS) (mg/l)	1751	1236	2320	1290	294	6620
Dissolved oxygen (ppm)	4,08	4,01	3,15	1,16	0,53	7,68
Na^+^ (mg/l)	279	190	90	266	20	1450
Ca^++^ (mg/l)	108	87	60	75	17	480
Mg^++^ (mg/l)	45	35	20	35	8	240
K^+^ (mg/l)	22,4	19,0	20,0	14,5	2,0	96,0
Chemical oxygen demand (COD) (mg/l)	8,4	6,0	0,5	9,5	0,5	49,0
Cl^-^ (mg/l)	503,5	294,4	209,5	537,9	44,3	2765,9
NO_3_^-^ (mg/l)	8,9	4,7	0,2	12,2	0,2	67,0
SO_4_^2-^ (mg/l)	102,2	70,3	48,0	99,0	7,3	632,2
HCO_3_^2-^ (mg/l)	293,6	292,8	219,6	91,1	91,5	579,5
Vital organism 22°C (UFC/ml)	101	44	12	162	1	1286
Vital organism 36°C (UFC/ml)	100	46	16	160	1	1440

**Figure 1:**
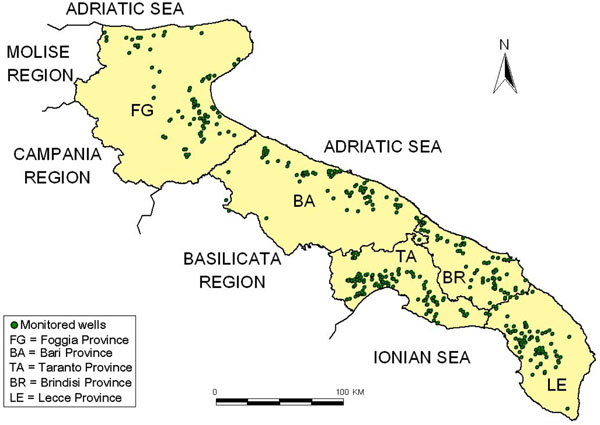
Water quality monitoring sites in Apulia region.

In figure 1 it’s possible to see the sampling sites (green dots) displaced in the five provinces of the Apulia region. Moreover in the figure 1 we can see the frontiers of Apulia region: Adriatic sea, Ionic sea, Basilicata region, Campania region and Molise region.

**Figure 2:**
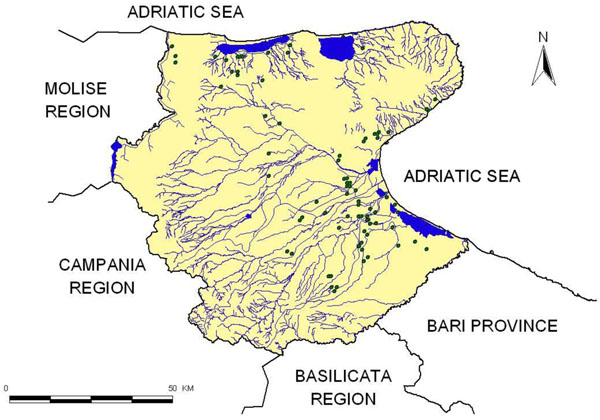
Foggia province with monitored wells.

In figure 2 the sampling sites (green dots) in Foggia province are pointed out, while the high amount of waterway, channels and lakes is shown in blue. In figure 2 the frontiers of Foggia province are also highlighted.

**Figure 3:**
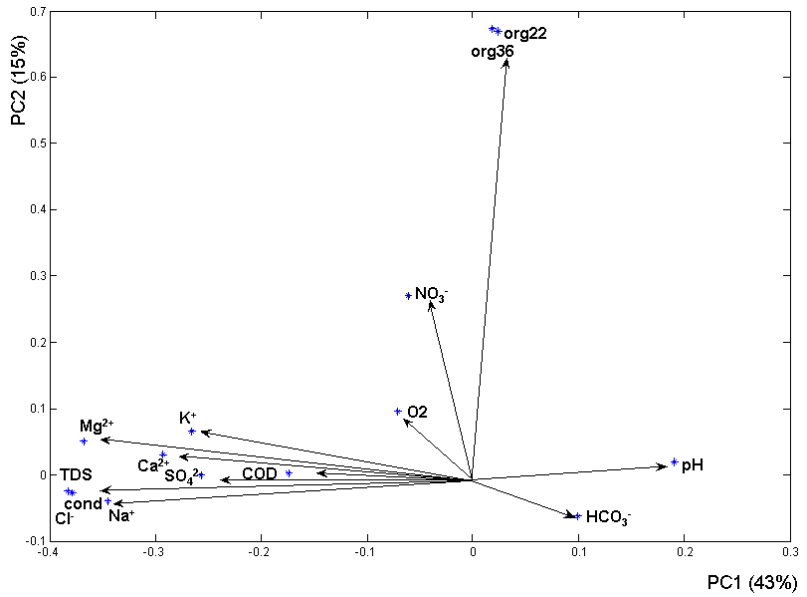
Principal component analysis loading plot.

In figure 3 loading plot for the data set analyzed is shown. It’s evident high loading values (negative in this case) for magnesium (Mg^2+^), potassium (K^+^), calcium (Ca^2+^), sulphate (SO_4_^2-^), total dissolved solids (TDS), Electrical Conductivity (cond) and sodium (Na^+^) on the first component explaining 43% of the total variance, while second component (explaining 18% of the total variance) has strong loadings on nitrate (NO_3_^-^), vital organism at 22°C and 36°C.

**Figure 4:**
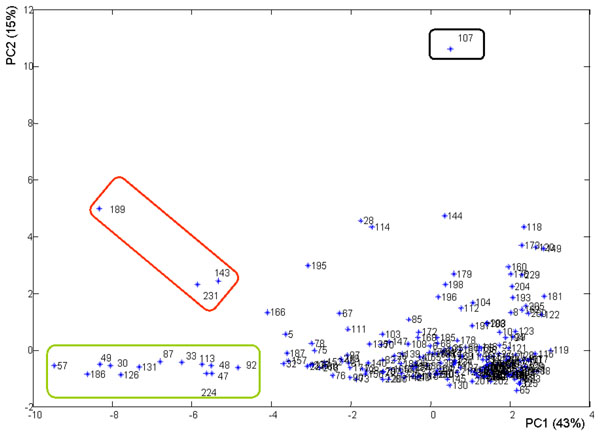
Principal component analysis score plot.

Considering the scores plot, shown in figure 4, in the plane of first and second Principal Component it is possible to note some scattered samples, highlighted in rectangular lines. The sample enclosed in the black line shows high loading values for parameters such as vital organism at 22 °C and 36°C and NO_3_^-^, samples enclosed in the red line show high loading values of Mg^2+^, K^+^, Ca^2+^and samples enclosed in the green line show high loading values of cond, Na^+^, chloride (Cl^-^), and TDS.

**Figure 5:**
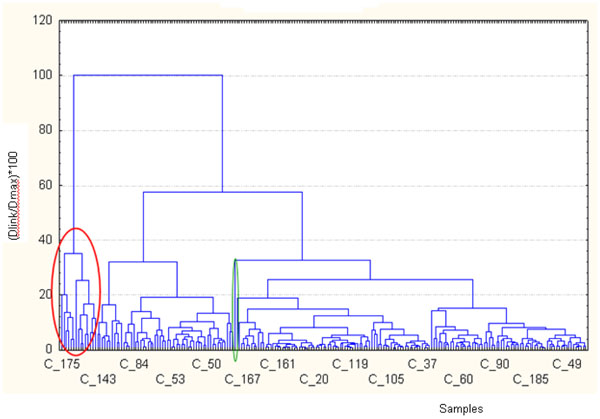
Cluster analysis tree diagram obtained from data set. On the x axis the sample numbers are displayed, while on the y axis the ratio Dlinkage/Dmax *100 is shown.

Applying a conglomeration hierarchic cluster method (complete linkage) to 219 cases we have obtained the dendrogramm shown in figure 5. The first cluster, highlighted by red circle, contains the samples that in the score plot (figure 4) scattered on the left of the plot, while the singleton highlighted in green is the sample number 107 that in the score plot scattered for vital organism at 22 and 36°C and NO_3_^-^.

**Figure 6:**
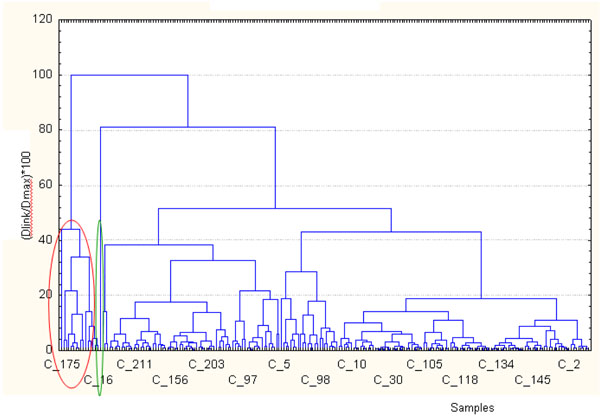
Cluster analysis tree diagram obtained from the first two component’s scores.

In figure 6 a clustering of the first two component’s scores is shown. We find that the first cluster contains the same samples of the previous dendrogram (figure 5) and the singleton is always the sample number 107.

In the dendrogram of figure 5 the similarity was measured keeping all the original information for each variable, also the noise. By using the scores of the first two components the similarity is linked to the meaning of the first two components.

The samples 143, 231, as the sample 189, highlighted in figure 4 with red circular line, scattered for high values of Mg^2+^, K^+^, Ca^2+^. These are typical cations of nutrients used as fertilizer in grain and tomatoes crops. The sites where these samples were collected were located in farms which main activities were grain and tomatoes crops.

**Figure 7:**
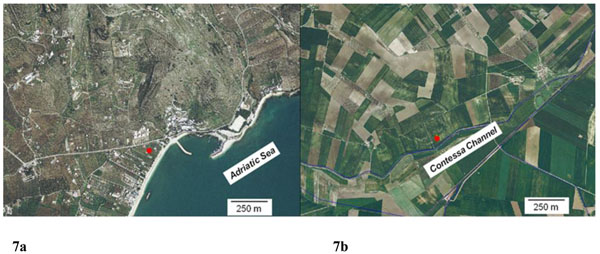
7a Orthophoto showing the sampling site located close to the sea in Mattinata village (Foggia province); 7b Orthophoto showing the sampling site close to *La Contessa* channel (Foggia province).

Other samples, such as the number 30, scattered for high values of cond, Cl^-^, Na^+^ and TDS. This sampling was performed after a summer season very dry. This means that an intrusion of marine water was in this site. The orthophoto (see figure 7a) supports this explanation showing that the site is very close to the sea. Several other samples, as highlighted in figure 4 with green circular line, show high values for Electrical Conductivity, Cl^-^, Na^+^ and TDS mostly. Also for these sites we can hypothesize an intrusion of marine water in the site: even if they are not too close to the sea, these sites are located in farms with high extension, so for these sites it’s been an overuse of the ground water.

On the contrary the sample number 107 shows a scattering for vital organism at 22 and 36 °C and NO_3_^-^ (see the black circular line in figure 4). The corresponded site is located along *La Contessa* channel, as one can see observing figure 7b. The sampling was performed on September 2007 during a period of time in which the water from *La contessa* channel was used for irrigation. In this channel waters from municipal purifier of Foggia city and waters from paper mill purifier pour. So irrigation water not well purified was used.

In fact organisms growing best at 36 °C, probably, come from external sources: they are bacteria belonging to the mesophilic flora derived from humans and animals. The colonies count at 36 °C increases, therefore, suspects of fecal pollution, reports undesirable changes and should lead to perform additional inspections. It’s an anthropogenic pollution index. The colonies count at 22 °C, although it does not have any health implication, allows us to highlight, in terms of quality and quantity, the putrefactive microbial species, spore-forming and chromogenic, abundant in the surface layers of soil and air, easily adaptable to the water environment. It’s an index of environmental pollution.

Form results obtained by Principal Component and cluster analyses it’s evident that some sampling sites investigated in Foggia district show dissimilarities, mostly due to the location of the site, the land use and management techniques and groundwater overuse. For all these reasons several natural and anthropogenic sources affect the groundwater quality of the investigated sites.

**Figure 8:**
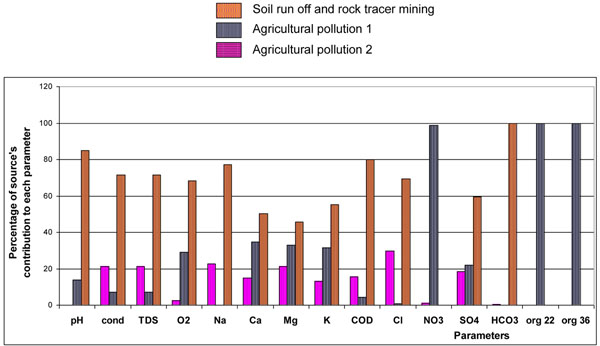
Percentage of source’s contribution to each parameters.

In order to individuate the pollutant sources the APCS method was applied to the data matrix of physical-chemical parameters collected. By APCS method it’s been possible to identify three pollutant sources. Observing figure 8, NO_3_^-^, org 22, org 36 are completely apportioned to a source named Agricultural pollution 1 due to fertilizer applications, both chemical and muck and use of not well purified effluent; Na^+^, Ca^2+^, Mg^2+^, K^+^, Cl^-^ ,SO_4_^2-^ are apportioned to source named Agricultural pollution 2 due to microelements for agriculture and groundwater overuse (Na^+^, Cl^-^); bicarbonate (HCO_3_^-^), chemical oxygen demand (COD), oxygen dissolved (O2), TDS, Cl^-^ are mostly apportioned to a source that can be identified as soil run off and rock tracer mining.

The weigh percentage of the sources are: 32% for agricultural pollution 1, 12% for agricultural pollution 2 and 56% for soil run off and rock tracer mining**.**

The error on the reconstructed concentration data matrix obtained by equation 2 was 3.2%.

## Experimental

### Data treatment and multivariate statistical methods

Table 3 shows the descriptive statistics used in this paper. For each parameter they are average, median, mode, standard deviation, minimum and maximum value.

Multivariate analysis of the groundwater data set was performed by PCA, CA and APCS. PCA and APCS elaborations were obtained by Matlab softwares (MATLAB 7.0) developed from authors. CA was performed by Statistica software (Stat Soft, version 8).

## Principal component analysis

PCA includes correlated variables with the purpose of reducing the numbers of variables and explaining the same amount of variance with fewer variables (principal components). The new variables created, the principal components scores (PCS), are orthogonal and uncorrelated to each other, being linear combinations of the original variables. They are obtained in such a way that the first PC explains the largest fraction of the original data variability, the second PC explains a smaller fraction of the data variance than the first one and so forth [14-16]. Varimax rotation is the most widely employed orthogonal rotation in PCA, because it tends to produce simplification of the unrotated loadings to easier interpretation of the results. It simplifies the loadings by rigidly rotating the PC axes such that the variable projections (loadings) on each PC tend to be high or low.

Generally two methods are used in order to chose p Eigenvectors: Kaiser method (PCs with eigenvalues greater than 1) and ODV70 ones (PCs representing at least 80% of the original data variance). In our method we have chosen the second one and we have taken into account p Eigenvectors until the sum of their Eigenvalues reaches at least 70% of the total variance.

## Cluster analysis

CA groups the objects (cases) into classes (clusters) on the basis of similarities within a class and dissimilarities between different classes. The results of CA help in interpreting the data and indicate patterns [12,17]. In hierarchical clustering, clusters are formed sequentially by starting with the most similar pair of objects and forming higher clusters step by step. Hierarchical agglomerative CA was performed on the data set by means of the Complete linkage’s method using squared Euclidean distances as a measure of similarity [18]. Cluster analysis was applied to the ground water data set with a view to group the similar sampling sites (spatial variability) spread over the Foggia province basin and in the resulted dendrogram, the linkage distance is reported as *D*link/*D*max, which represents the quotient between the linkage distance for a particular case divided by the maximal distance, multiplied by 100 as a way to standardize the linkage distance represented on *y*-axis [11,12,19].

## Absolute principal component scores

The reconstruction of the source profile and contribution matrices can be successfully obtained by APCS method [20,21].

In the APCS method the first step, that agrees with PCA, is the search of the Eigenvalues and Eigenvectors of the data correlation matrix G. Only the most significant p Eigenvectors (or factors) are taken into account. In our method we have taken into account p Eigenvectors until the sum of their Eigenvalues reaches at least 70% of the total variance.

The p Eigenvectors are then rotated by an orthogonal or oblique rotation. The most used rotation algorithm is Varimax, which performs orthogonal rotation of the loadings. After the rotation all the components should assume positive values; small negative values are set zero. An abstract image of the source contributions to the samples can be obtained by the following multivariate linear regression:(1)

where Z is the scaled data matrix, PCS is the principal component scores matrix, and V^T^ is the transposed rotated loading (Eigenvectors) matrix.

In order to pass from the abstract contributions to real ones, a fictitious sample Z0, where all concentrations are zero, is built [20,22].

Using the matrix V^T^ and the Equation (1) the vector PCS0, corresponding to Z0, is calculated and subtracted from all the vectors that form PCS. The matrix obtained in this way is referred to as Absolute Principal Component Scores (APCS) matrix. The APCS matrix can be identified with the estimated contribution matrix (F_r_). Also in this case small negative values are usually set zero. Then, a regression on the data matrix X allows to obtain the estimated source profiles matrix (A_r_). If the APCS matrix is bordered with a unit column vector, the regression gives for each parameter also a possible contribution of the not explained variance.

At last the product of the matrices F_r_ and A_r_ allows to recalculate the data matrix (X_r_).

If F and A are unknown, the agreement between X and X_r_ is the only assessment for the effectiveness of their reconstruction.

An error measure that better determines the mean error on the data is defined as:(2)

where normf is the Frobenius’s norm and X_r_ is the data matrix reconstructed and X is the data matrix, respectively.

## Conclusions

Multivariate statistical methods represent a valid tool to understand complex nature of groundwater quality issues, determine priorities in the use of ground waters as irrigation water and suggest interactions between land use and irrigation water quality. The results obtained by multivariate statistical methods can be used to suggest to stakeholders, for example, a mitigation in the groundwater overuse of some wells mostly in dry seasons and to require orderly quality tests of the channel waters when they are used for crop irrigation.

## Methods

### Sampling

The sampling sites have been identified taking into account the main type of land use and their management techniques in each Apulian province, as indicated below:

1. Olive, vine and cherry for Bari province;

2. Olives, grapes, and tomatoes for Brindisi;

3. Olives, grapes, tomatoes and wheat for Foggia;

4. Olive, tomato and citrus for Lecce;

5. Olive, vine and citrus for Taranto.

The wells monitored during the sampling campaign have been individuated inside specific farms which adopted agricultural practices (crops, tillage, irrigations, fertilizations, pesticides applications, etc) have been considered, according to the official agricultural statistics, representative of the usual land management. In table 4 the number of monitored wells and collected samples for each Apulian province are shown.

**Table 4:** Groundwater quality monitoring

PROVINCE	WELLS	SAMPLES
BARI	96	261
BRINDISI	89	104
FOGGIA	85	219
LECCE	84	166
TARANTO	119	259

These farms also, according to an agreed protocol, had to present these specific characteristics:

1. to have a continuous crops area ≥ 1.0 ha;

2. to use for irrigation only water coming from the monitored wells;

3. to have the land management register regularly compiled in the last two years;

4. to be close (≤ 5.0 km) to a meteorological gauge-station.

In this paper we show the results obtained from monitoring activity performed in Foggia province. The amount of monitored wells were 85 and the total number of samples collected were 219.

The Province of Foggia (Area**:** 6,965 km² ; Population**:** about 680,000 inhabitants), placed in the South of Italy, is part of Apulia Region. It can be sub-divided in three geographic sub-regions: *Gargano* (the limestone mountains placed in the east part of the province), the *Tavoliere* (the alluvial plain placed in the central zone) and *Sub-Appennino* (the mountainous system in the west part). A complex river network characterizes Sub-Appennino and Tavoliere, where several streams flow from west to east. The main rivers are *Fortore* (in the north, along the boundary with the Molise region), *Candelaro* (which separates Tavoliere from Gargano) and *Cervaro* (in the South).

The province of Foggia is one of the most important agricultural areas of Italy, especially its alluvial plain. The main crops are winter wheat, tomatoes, vegetables, orchard, vineyard and olive groves.

Samples from the boreholes were collected using manually operated hand pumps.

Sampling took place under dynamic conditions, after flushing a large amounts of water for about 30 minutes.

All samples were kept in two liters polyethylene bottles, which have been previously washed with 1:1 HCl and distillated water. The bottles, which have cap and under cap, were filled to the brim in order to prevent the transfer of the analytes in the headspace and their loss at the opening of the bottles.

After collection, samples were stored in cooled bags and transported to the laboratory as soon as possible.

They were stored in the refrigerator at about 4°C before the analysis without chemical preservatives because the analysis was performed either directly on-site, or immediately in the laboratory.

## Sample analyses

The samples were analyzed for pH, Electrical Conductivity, TDS, O_2_, COD, the major ions (ie. Na^+^, Ca^2+^, Mg^2+^, K^+^, Cl^-^, NO_3_^-^, SO_4_^2-^ and HCO_3_^-^), vital organism at 22 and 36 °C.

The chemical and physical analyses of water samples have been carried out according to the official guideline proposed by the Ministero delle Politiche Agricole (the national agriculture authority) in a specific law (Decreto Ministeriale del 23 Marzo 2000 “*Metodi ufficiali di analisi delle acque per uso agricolo e zootecnico*” [23]).

Some physical-chemical parameters such as pH, Electrical Conductivity, TDS and dissolved oxygen were determined immediately after sampling. All field meters were checked and calibrated according to the manufacturer’s specifications.

In particular, the pH meter (Hanna instruments, model 9025) was calibrated using two buffers of pH 7.0 and 10.0.

Conductivity /TDS meter (Hanna Instruments, model 9835) was used to measure the conductivity and total dissolved solids of the water samples. The instrument was calibrated with 0.001M KCl to give a value of 14.7 μS/m at 25°C. The probe was thoroughly rinsed with distilled water after each measurement.

Dissolved oxygen meter (Hanna Instruments, model 9143) was automatically standardized to the actual saturation value (after setting the appropriate working altitude) prior to each measurement set.

Chemical parameters were determined after filtration of the sample under vacuum on cellulose acetate filters with porosity of 0,45 microns.

About COD measurements, 2.0 mL of sample was added to a vial (sample vial) filled by the manufacturers with the reagent solution (HI 93754A-25 for a low range of COD: 0 – 150 mg/L). 2.0 mL of deionized water was added to other vial (blank vial). The vials was heated for 2 hours at 150°C. During this digestion period oxidizable organic compounds reduce the dichromate ion (orange) to the chromic ion (green). The amount of remaining dichromate was automatically determined with a multiparameter bench photometer (Hanna Instruments C99).

The samples were analyzed for Na^+^, Ca^2+^, Mg^2+^, K^+^ using a Varian atomic absorption spectrophotometer (model SpectrAA – 250 PLUS).

The anions Cl^-^, NO_3_^-^, SO_4_^2-^ were measured by ion chromatography (Dionex corporation, model AS50) and bicarbonate^-^ by a simple alkalimetric method.

The performance of spectrophotometer (cations determination) and ion chromatography (anions determination) was checked by passing standard solutions of all measured parameters. Blank samples (deionized water) were analyzed after every six measurements of water samples to check for any eventual contamination or abnormal response of equipment.

For the control of the quality of the analytical results, the ion balance was computed by summing up the equivalent concentrations of cations and anions of the samples. The sum of anion equivalent concentrations  should be equal to the cations  ones according to the neutrality condition:(3)

The colonies count at 36 °C and 22 °C (vital organism at 36 °C and 22 °C) is considered an indicator of poor protection of a hydric environment. The use of different temperatures highlights mesophilic microorganisms (36 °C) and psychrophilic (22 °C).

In the analytical method [24] used in this paper for the vital organism at 22 °C and 36 °C determination, Plate Count Agar (PCA), a microbiological growth medium agar, no selective, enriched with tryptone, yeast extract and glucose, which allows growth of almost all undifferentiated microbial species in the water sample, was used.

## Competing interests

The authors declare that they have no competing interests.

## Authors’ contributions

PI: Statistical analysis, elaborations and results interpretation. Drafting manuscript. DC: Water physical-chemical analysis. Results interpretation. Contribution to drafting manuscript AL: Coordination of the study and contribution to drafting manuscript. PAD: Water physical-chemical analysis. GP: GIS elaborations. Results interpretation. VFU: Coordination of the project and participation in its design.

## Funding

This study was supported by the “Expansion of regional agro-meteorological network” project funded by Apulia Region (Italy).

## References

[B1] Schot PP van der Wal J Human impact on regional groundwater composition through intervention in natural flow patterns and changes in land use J Hydrol 1992 134 1-4 297 313 10.1016/0022-1694(92)90040-3

[B2] Kannel PR Lee S Lee YS Assessment of spatial–temporal patterns of surface and ground water qualities and factors influencing management strategy of groundwater system in an urban river corridor of Nepal J Environ Manage 2008 86 4 595 604 10.1016/j.jenvman.2006.12.021 17287068

[B3] Singh KP Malik A Sinha S Water quality assessment and apportionment of pollution sources of Gomti river (India) using multivariate statistical techniques - a case study Anal Chim Acta 2005 538 1-2 355 374 10.1016/j.aca.2005.02.006

[B4] Dixon W Chiswell B Review of aquatic monitoring program design Water Res 1996 30 9 1935 1948 10.1016/0043-1354(96)00087-5

[B5] Debels P Figueroa R Urrutia R Barra R Niell X Evaluation of water quality in the Chillán River (Central Chile) using physicochemical parameters and a modified water quality index Environ Monit Assess 2005 110 1-3 301 322 10.1007/s10661-005-8064-1 16308794

[B6] McGarigal K Cushman S Stafford S Multivariate Statistics for Wildlife and Ecology Research 2000 New York: Springer

[B7] Simeonova P Simeonov V Andreev G Water quality study of the Struma River Basin, Bulgaria Cent Eur J Chem 2003 1 2 121 136 10.2478/BF02479264

[B8] Simeonov V Simeonova P Tsitouridou R Chemometric quality assessment of surface waters: two case studies Ecol Chem Eng 2004 11 6 450 469

[B9] Bengraine K Marhaba TF Using principal component analysis to monitor spatial and temporal changes in water quality J Hazard Mater 2003 100 1-3 179 195 10.1016/S0304-3894(03)00104-3 12835021

[B10] Liu CW Lin KH Kuo YM Application of factor analysis in the assessment of groundwater quality in a blackfoot disease area in Taiwan Sci Total Environ 2003 313 1-3 77 89 10.1016/S0048-9697(02)00683-6 12922062

[B11] Simeonov V Stratis JA Samara C Zachariadis G Voutsa D Anthemidis A Sofoniou M Kouimtzis Th Assessment of the surface water quality in Northern Greece Water Res 2003 37 17 4119 4124 10.1016/S0043-1354(03)00398-1 12946893

[B12] Singh KP Malik A Mohan D Sinha S Multivariate statistical techniques for the evaluation of spatial and temporal variations in water quality of Gomti River (India)-a case study Water Res 2004 38 18 3980 3992 10.1016/j.watres.2004.06.011 15380988

[B13] European Environment Agency (EEA) CORINE Land Cover Project 2005

[B14] Abdul-Wahab SA Bakheit CS Al-Alawi SM Principal component and multiple regression analysis in modelling of ground-level ozone and factors affecting its concentrations Environ Modell Softw 2005 20 10 1263 1271 10.1016/j.envsoft.2004.09.001

[B15] Sousa SIV Martins FG Alvim-Ferraz MCM Pereira MC Multiple linear regression and artificial neural networks based on principal components to predict ozone concentrations Environ Modell Softw 2007 22 1 97 103 10.1016/j.envsoft.2005.12.002

[B16] Wang S Xiao F AHU sensor fault diagnosis using principal component analysis method Energ Buildings 2004 36 2 147 160 10.1016/j.enbuild.2003.10.002

[B17] Vega M Pardo R Barrado E Deban L Assessment of seasonal and polluting effects on the quality of river water by exploratory data analysis Water Res 1998 32 12 3581 3592 10.1016/S0043-1354(98)00138-9

[B18] Todeschini R Introduzione alla chemiometria 1998 Napoli: EdiSES s.r.l.

[B19] Wunderlin DA Díaz MDP Amé MV Pesce SF Hued AC Bistoni MdlA Pattern recognition techniques for the evaluation of spatial and temporal variations in water quality. A case study: Suquía River Basin (Córdoba–Argentina) Water Res 2001 35 12 2881 2894 10.1016/S0043-1354(00)00592-3 11471688

[B20] Thurston GD Spengler JD A quantitative assessment of source contributions to inhalable particulate matter pollution in metropolitan Boston Atmos Environ 1985 19 1 9 25 10.1016/0004-6981(85)90132-5

[B21] Caselli M de Gennaro G Ielpo P A comparison between two receptor models to determine the source apportionment of atmospheric pollutants Environmetrics 2006 17 5 507 516 10.1002/env.788

[B22] Ielpo P Heavy metals and atmospheric particulate: chromatographic analysis, scanning electron microscopy and source apportionment PhD thesis 2004 University of Bari, Italy (Stored to the Public Libraries of Rome and Florence - BNI0013860- Italy)

[B23] Decreto Ministeriale del 23 Marzo 2000 Approvazione dei “Metodi ufficiali di analisi delle acque per uso agricolo e zootecnico” 2000

[B24] APAT CNR IRSA 7050 Manual 29 2003

